# MRI Evidence: Acute Mountain Sickness Is Not Associated with Cerebral Edema Formation during Simulated High Altitude

**DOI:** 10.1371/journal.pone.0050334

**Published:** 2012-11-30

**Authors:** Klemens Mairer, Markus Göbel, Michaela Defrancesco, Maria Wille, Hubert Messner, Alexander Loizides, Michael Schocke, Martin Burtscher

**Affiliations:** 1 Department of Sports Science, Medical Section, University of Innsbruck, Innsbruck, Austria; 2 Department of Psychiatry and Psychotherapy, University Hospital, Medical University Innsbruck, Innsbruck, Austria; 3 Department of Radiology, University Clinic of Radiology I, Medical University Innsbruck, Innsbruck, Austria; University of Las Palmas de Gran Canaria, Spain

## Abstract

Acute mountain sickness (AMS) is a common condition among non-acclimatized individuals ascending to high altitude. However, the underlying mechanisms causing the symptoms of AMS are still unknown. It has been suggested that AMS is a mild form of high-altitude cerebral edema both sharing a common pathophysiological mechanism. We hypothesized that brain swelling and consequently AMS development is more pronounced when subjects exercise in hypoxia compared to resting conditions. Twenty males were studied before and after an eight hour passive (PHE) and active (plus exercise) hypoxic exposure (AHE) (F_i_O_2_ = 11.0%, P_i_O_2_∼80 mmHg). Cerebral edema formation was investigated with a 1.5 Tesla magnetic resonance scanner and analyzed by voxel based morphometry (VBM), AMS was assessed using the Lake Louise Score. During PHE and AHE AMS was diagnosed in 50% and 70% of participants, respectively (p>0.05). While PHE slightly increased gray and white matter volume and the apparent diffusion coefficient, these changes were clearly more pronounced during AHE but were unrelated to AMS. In conclusion, our findings indicate that rest and especially exercise in normobaric hypoxia are associated with accumulation of water in the extracellular space, however independent of AMS development. Thus, it is suggested that AMS and HACE do not share a common pathophysiological mechanism.

## Introduction

Acute mountain sickness (AMS) constitutes a widespread medical condition among un-acclimatized individuals ascending to high altitude (>2500 m) too fast. Symptoms include headache, being the key symptom of AMS, gastrointestinal complaints, vomiting, weakness, lethargy and insomnia, typically developing within 6–10 hours after arrival at high altitude [1]. Despite intense research activities in this field [2]–[4] exact mechanisms causing the symptoms of AMS remain elusive. A prevailing hypothesis however, points to a pathophysiological process within the central nervous system suggesting that AMS is a self-limiting mild form of HACE (high altitude cerebral edema) both sharing a common pathophysiological mechanism. Thus, AMS may be considered an early stage of subclinical brain edema leading to intracranial hypertension and the symptoms of AMS [5]. This hypothesis has been supported by two recently published magnetic resonance imaging (MRI) studies (1.5 and 3.0 Tesla) which provided convincing evidence for mild vasogenic brain swelling in subjects with (AMS+) and without AMS (AMS−) following 6–18 h passive exposure to normobaric hypoxia (F_i_O_2_∼12%). In contrast, cytotoxic (intracellular) edema was detected only in AMS+ subjects, indicated by a decreased apparent diffusion coefficient (ADC) [3], [6].

Strenuous exercise during high altitude exposures seems to exaggerate the effects of hypoxia, thereby increasing AMS prevalence and severity as previously reported by Roach et al. [7]. They demonstrated that exercise per se resulted in a higher AMS prevalence and severity at a simulated altitude of 4800 m. Similar findings are reported by Burtscher et al. [8] who found a substantially higher incidence of high altitude headache (HAH) when exercising at high altitude compared to resting conditions. This relationship has recently been confirmed by two observational studies [9], [10]. Therefore, we hypothesized that brain swelling and consequently AMS development is more pronounced when subjects exercise in hypoxia compared to resting conditions. Thus, the aim of the present study was to investigate the formation of potential brain edema and related AMS development during rest and exercise in normobaric hypoxia. The method of Voxel based morphometry was used in order to measure changes of gray and white matter volume and ADC values of the entire brain.

## Materials and Methods

### Subjects

Twenty young and healthy male volunteers were recruited for the present study. Exclusion criteria were an overnight stay at altitudes above 2500 m in the previous two months [11], permanent residence above 1000 m, a history of cardiopulmonary, neurological and psychiatric diseases, chronic headache, a history of migraine or contraindications to undergo MRI (e.g. cardiac pace maker). Although subjects did not regularly consume coffee, they were asked to abstain from caffeinated beverages and alcohol and to avoid intense physical exercise 24 h prior to the study. Characteristics of all participants are presented in Table 1. The present study was approved by the ethic committee of the Medical University of Innsbruck and was conducted in accordance with the Declaration of Helsinki of the World Medical Association. Written informed consent was obtained from all subjects prior to the participation in the study.

**Table 1:** Characteristics of all study participants and separated for subjects with (AMS+) and without AMS (AMS−) during the passive (PHE) and active hypoxic exposure (AHE).

		Passive hypoxic exposure (PHE)			Active hypoxic exposure (AHE)		
	All subjects (N = 20)	AMS− (N = 10)	AMS+ (N = 10)	p^a^	AMS− (N = 6)	AMS+ (N = 14)	p^b^
Body mass, kg	76.0±8.2	77.3±5.8	74.7±9.2	0.49	77.0±4.2	75.6±9.6	0.65
Body height, cm	182.4±7.1	184.1±5.9	180.7±8.0	0.29	182.0±3.6	182.6±8.2	0.87
Age, yrs	28.3±3.7 (21–35)	27.9±3.7 (21–34)	28.6±3.8 (22–35)	0.68	27.3±4.2 (22–34)	28.7±3.6 (21–35)	0.48
Exercise training, h/week	8.3±3.1	9.0±3.7	7.5±2.2	0.30	9.7±2.3	7.7±3.3	0.19
Altitude of permanent residence, m	486±200	521±216	452±188	0.46	531±207	467±202	0.53
History of AMS, yes/no	3/17	1/9	2/8	0.53	1/5	2/12	0.89
Prior altitude experience, yes/no	15/5	8/2	7/3	0.60	5/1	10/4	0.57
Smoking, yes/no	0/20	0/10	0/10	-	0/6	0/14	-
Use of drugs yes/no	0/20	0/10	0/10	-	0/6	0/14	-

p^a^ and p^b^: differences between AMS− and AMS+ subjects during PHE and AHE, respectively. Data are expressed as means ± standard deviation (range) or as frequencies.

### Study design

A randomized crossover study design was applied. Subjects were randomly assigned to either the passive hypoxic exposure (PHE) or the active (plus exercise) hypoxic exposure (AHE). After an interval of at least four weeks participants returned to complete the second protocol, vice versa to the initial allocation.

Baseline examinations included MR imaging, measurements of heart rate (HR), arterial oxygen saturation (SpO_2_) for 5 min, blood pressure (BP), assessment of acute mountain sickness (AMS) and a short clinical routine examination and were performed at an altitude of 575 m (Innsbruck, Austria) in the morning hours of the respective hypoxic exposure.

The subsequent hypoxic trial involved an 8 hour exposure to normobaric hypoxia in a hypoxic chamber (hypoxic room systems Hypoxico OHG, Germany) at an inspired oxygen fraction (F_i_O_2_) of 11.0% which equates to a terrestrial altitude of ∼5500 m (based on the altitude of Innsbruck: 575 m). The PHE was spent sedentary, while the AHE included three 30-min exercise periods in hypoxia (ExH) at 50% of the subjects' individual maximal altitude-specific exercise performance (Watt_max_). During PHE and AHE, measurements of HR, SpO_2_, Bp and AMS were accomplished after 2, 4, 6 and 8 hours, as described in “baseline examinations”. Food and drinks were provided and could be consumed ad libitum. Ambient F_i_O_2_ and inspired fraction of carbon dioxide (F_i_CO_2_) were kept at 11.1±0.4% and 0.8±0.3% during PHE and 11.2±0.4 and 0.8±0.2 during AHE, respectively.

MR imaging was repeated immediately after the hypoxic trials. Patients were transported to the magnetic scanner within several minutes in normoxia. MR examinations started on average 10 to 15 minutes after the end of the hypoxic exposures.

To determine the individual maximal altitude-specific exercise performance (Watt_max_) for ExH, each subject had to complete an incremental exercise test until exhaustion on a programmable, electrically braked cycle ergometer (Ergometrics 900, Ergoline, Bitz, Germany). The cycle ergometry was performed at a simulated altitude of 5500 m (F_i_O_2_ = 11.0%) at least eight weeks prior to the study. After a warm-up period of 3 min duration at 50 Watt, power output was increased by 50 Watt every third minute until volitional exhaustion or until the subjects were unable to maintain a pedaling rate of 50 min^−1^. A test was considered maximal if both of the following criteria were met: HR_max_≥90% of age predicted maximum HR (220 – age) and a rate of perceived exertion (RPE)≥19 (BORG-RPE scale) at maximal exercise.

### Data acquisition

#### Magnetic Resonance Imaging (MRI)

Using a 1.5 Tesla whole-body MR scanner (Magnetom Avanto, Siemens Erlangen, Germany) and an eight-channel head coil, all volunteers underwent MR imaging of the brain. The MR protocol comprised following sequences:

- Coronal T1-weighted MPRAGE 3D, field of view (FoV) 220×165 mm, slice thickness (SLT) 1.2 mm, repetition time (TR) 1600 ms, inversion time (TI) 800 ms, echotime (TE) 3.44 ms, matrix 256×192, iPAT factor 2, 160 slices
- Transversal diffusion-weighted echoplanar imaging, FoV 230×230 mm, SLT 3 mm, 35 slices, TR 6400 ms, TE 99 ms, matrix 128×128, iPAT factor 2, b-values 0 and 1000 mm/s^2^, diffusion-sensitizing gradients in 12 directions

Diffusion-weighted images were aligned parallel to the AC-PC line and comprised between 37 and 41 slices in order to cover the whole brain. Maps of mean diffusivity and fractional anisotropy were calculated by fitting the signal of the diffusion-weighted images.

### Arterial oxygen saturation (SpO_2_)

SpO_2_ was monitored by fingertip pulse oximetry (Pulsox-3i, Minolta, Osaka, Japan) for 5 min, while the subjects were in a sitting position and were instructed to breathe regularly and quietly.

### Acute mountain sickness (AMS)

Presence and severity of AMS was determined using the Lake Louise Score (LLS) [12]. The self-report section of the LLS contains five multiple choice questions inquiring after severity of headache, gastrointestinal symptoms like anorexia, nausea or vomiting, fatigue and/or weakness, dizziness and/or lightheadedness and quality of sleep, which are rated by the subjects from 0 - no discomfort to 3 - severe symptoms. Due to the fact that our subjects did not sleep in the hypoxic chamber, the symptom complex “quality of sleep” was excluded. The LLS is obtained by adding all points of the self-report section. A diagnosis of AMS is based on the presence of headache, at least one additional symptom and a total LL score of ≥4 [13].

### Image processing and statistical analysis

T_1_ and diffusion weighted images were processed using SPM8 software (Wellcome Department of Imaging Neuroscience Group, Institute of Neurology, London) running with MATLAB 7.11 R2010b (Mathworks Inc., Natick, MA, USA). First, the raw data in DICOM (.dcm) format were imported into SPM. In the next step, T1 weighted images were spatially normalized to the standard MNI template of the Montreal Neurological Institute (MNI) and segmented into grey matter (GM), white matter (WM) and cerebrospinal fluid (CSF) using the SPM8 unified segmentation with default settings [14]. The gray and white matter maps were then spatially normalized to a group specific template (composed of all subjects) in MNI space using the DARTEL toolbox [15]. The resulting maps were smoothed with an 8 mm full-width half maximum (FWHM) Gaussian Kernel to increase the signal to noise ratio before statistical processing. Due to the fact that ADC values do not compartmentalize in GM and WM, analysis on the whole brain were conducted without segmentation. All diffusion weighted images were in a first step co-registered with the corresponding high resolution T1 images. Next, ADC volumes were spatial normalized to MNI space by applying the warps of the T1 image. Finally, these normalized ADC data were smoothed with an 8 mm (FWHM) Gaussian kernel. Quality controls were performed after every step by visual inspection.

Voxel-wise comparison between baseline and follow up measurements was performed using a paired t-test, differences between groups (AMS− and AMS+) were analyzed using an unpaired t-test. *P*-values less than 0.001, uncorrected, were considered statistically significant. The graphical presentation of MR images was performed with xjView 8, a viewing program for Statistical Parametric Mapping.

### Statistical analysis

Clinical data are expressed as means ± standard deviation (SD) or frequencies (%). The Kolmogorov-Smirnov-test was applied to assess the distribution normality of the variables. An unpaired t-Test was used to analyze group differences (AMS+ and AMS−), for the comparison between two groups (PHE and AHE) a paired t-Test was applied. The association between continuous variables was determined using the Pearson correlation analysis. Qualitative and non-normally distributed data were analyzed with non-parametric statistics. Statistical difference was defined at an α level of *p*<0.05. Data were analyzed using SPSS (18.0) statistical-software package.

## Results

### Prevalence of AMS

All subjects completed the study. Ten subjects (50%) developed AMS during PHE and 10 had only minor symptoms that did not fulfill the criteria for AMS (headache and a LLS≥4). After 8 h in PHE AMS− and AMS+ subjects had LL scores of 1.7±1.1 and 5.9±1.4, respectively (p<0.001). During AHE the proportion of subjects who developed AMS was 70%, which was not statistically different from PHE (p = 0.22). LL scores of AMS− and AMS+ subjects during AHE amounted to 2.5±0.8 and 6.3±2.3, respectively (p<0.001). All subjects who developed AMS during PHE also got sick during AHE. Values of SpO_2_, HR and BP did not differ between subjects with and without AMS (Table 2).

**Table 2:** Oxygen saturation (SpO_2_) in %, heart rate (HR) in beats per min, systolic (Sys. Bp) and diastolic (Dia. Bp) blood pressure in mmHg of baseline measurements and during hypoxia.

	Passive hypoxic exposure (PHE)						Active hypoxic exposure (AHE)					
	BL_PHE_ (N = 20)	Hypoxia_PHE_ (N = 20)	p^a^	AMS− (N = 10)	AMS+ (N = 10)	p^b^	BL_AHE_ (N = 20)	Hypoxia_AHE_ (N = 20)	p^a^	AMS− (N = 6)	AMS+ (N = 14)	p^b^
**SpO_2_**	97.3±0.6	73.6±5.0	<0.001	72.8±2.9	75.6±7.2	0.27	97.3±0.8	74.0±6.0	<0.001	78.6±2.7	78.4±5.6	0.95
**HR**	66.4±9.1	79.2±11.5	<0.001	80.7±13.5	79.3±10.5	0.81	67.8±10.4	91.0±11.3	<0.001	82.4±3.7	89.7±12.0	0.17
**Sys. Bp**	125.1±11.6	116.4±7.8	<0.01	116.7±8.3	116.0±7.6	0.85	123.1±9.1	116.3±11.4	0.03	120.5±10.4	115.9±10.0	0.39
**Dia. Bp**	79.3±4.2	74.0±7.4	0.004	74.0±7.8	74.0±7.5	0.99	80.1±7.3	71.2±7.3	0.001	76.9±6.2	70.7±5.6	0.07

BL_PHE_ and BL_AHE_: Baseline measurements of the passive hypoxic exposure (PHE) and the active hypoxic exposure (AHE) of the whole group, Hypoxia_PHE_ and Hypoxia_AHE_: measurements during PHE and AHE (average of H2–H8) of the whole group. AMS+ and AMS−: subject with and without AMS. p^a^: difference between BL_PHE_ and Hypoxia_PHE_ respectively BL_AHE_ and Hypoxia_AHE_, p^b^: difference between AMS− and AMS+ subjects during PHE respectively AHE. Values are expressed as means ± standard deviation.

### Magnetic resonance imaging

#### Passive hypoxic exposure

**Figure 1:**
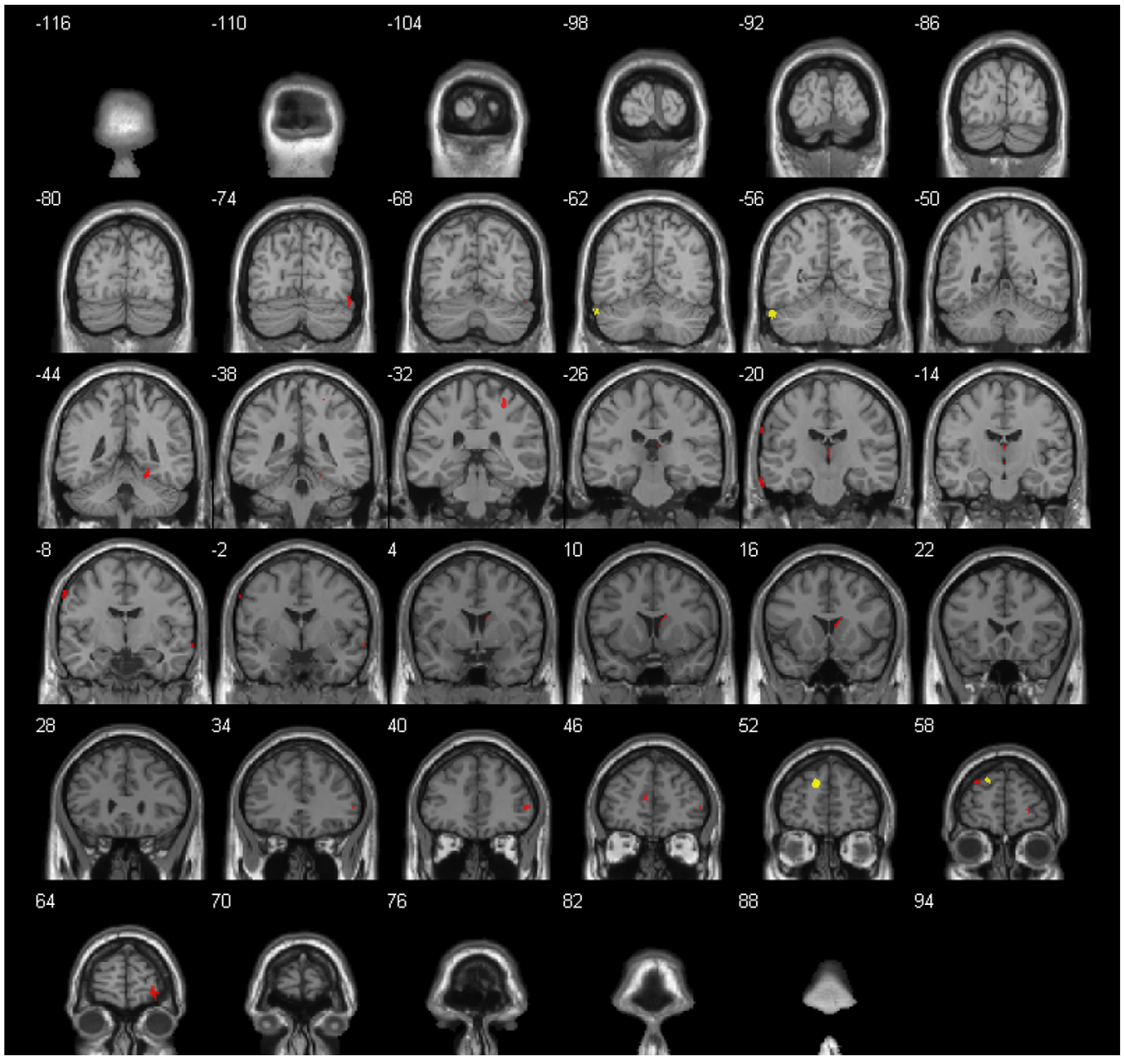
Regional cerebral changes during the passive hypoxic exposure (PHE) for the entire group (N = 20). Areas with significant increases of gray (GM) and white matter (WM) volume are presented in yellow, ADC increases are presented in red (p<0.001, uncorrected). Right hemisphere in the figure denotes left hemisphere of the brain and vice versa. A general T_1_ image provided by xjView 8 was used as background.

Results of VBM revealed an increase of white matter (WM) volume in the frontal lobe as well as of gray matter volume (GM) in the posterior lobe of the cerebellum after 8 hours of passive hypoxia (PHE) when compared to baseline measurements. Additionally, the frontal lobe, parietal lobe, temporal lobe, limbic lobe, the anterior lobe of the cerebellum and the sub-lobar region showed significantly higher ADC values during PHE when compared to baseline measurements. No significant hypoxia-induced decreases in GM and WM volume or ADC values were observed throughout the entire brain during PHE. Subjects with and without AMS did not differ regarding GM and WM volumes or ADC values. When subjects were classified in groups with (headache score ≥1) and without headache (headache score = 0), no differences in T_1_ or diffusion weighted images could be detected either. Tables 3 and 4 and Figure 1 summarize the GM, WM and ADC changes for the whole group caused by PHE.

**Table 3:** Anatomical regions with significant gray (GM) and white matter (WM) volume increases during the passive hypoxic exposure (PHE) (p<0.001, uncorrected).

Cerebral region		Matter	Side	Cluster size mm3	MNI coordinates	t	Z-score
**Cerebrum**							
Frontal Lobe	Superior Frontal Gyrus	WM	L	188	−10.5 54 25.5	5.31	4.02
**Cerebellum**							
Posterior Lobe	Tuber	GM	L	139	−57 −58.5 −31.5	4.55	3.63

Left (L), Right (R), coordinates (x, y, z) are given in Montreal Neurological Institute (MNI) space showing the center of each cluster.

**Table 4:** Anatomical regions with significant increases of apparent diffusion coefficient (ADC) during the passive hypoxic exposure (PHE) (p<0.001, uncorrected).

Cerebral region		Matter	Side	Cluster size mm^3^	MNI coordinates	t	Z-Score
**Cerebrum**							
Frontal Lobe	Precentral Gyrus		L	30	−62 −8 44	5.94	4.31
	Inferior Frontal Gyrus	GM	R	25	52 40 4	5.11	3.92
	Superior Frontal Gyrus	GM	L	29	−28 56 32	4.85	3.79
	Superior Frontal Gyrus	GM	R	45	28 64 −6	4.83	3.78
Parietal Lobe	Postcentral Gyrus	WM	R	29	28 −34 58	5.39	4.06
Temporal Lobe	Fusiform Gyrus	GM	R	24	52 −70 −18	4.93	3.83
	Middle Temporal Gyrus	GM	R	21	64 2 −10	4.39	3.54
Limbic Lobe	Anterior Cingulate	WM	L	20	−6 48 12	5.67	4.19
Sub-lobar	Extra-Nuclear	GM	R	39	10 16 14	5.15	3.94
	Caudate	WM	R	39	4 −24 14	5.00	3.87
**Cerebellum**							
Anterior Lobe	Culmen		R	36	22 −44 −16	5.48	4.10

Gray matter (GM), white matter (WM), Left (L), Right (R), coordinates (x, y, z) are given in Montreal Neurological Institute (MNI) space showing the center of each cluster.

#### Active hypoxic exposure

**Figure 2:**
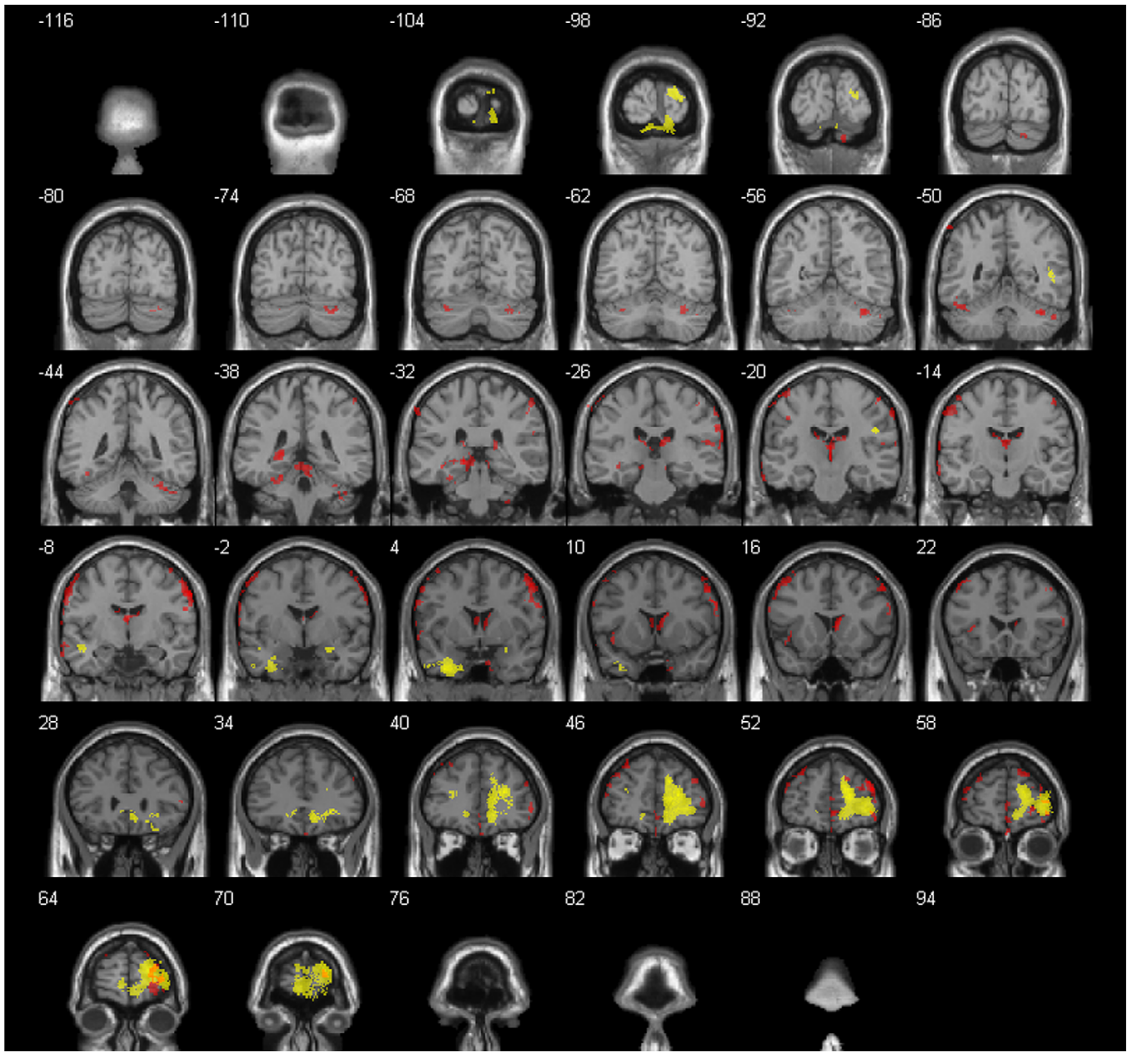
Regional cerebral changes during the active hypoxic exposure (AHE) for the entire group (N = 20). Areas with significant increases of gray (GM) and white matter (WM) volume are presented in yellow, ADC increases are presented in red (p<0.001, uncorrected). Right hemisphere in the figure denotes left hemisphere of the brain and vice versa. A general T_1_ image provided by xjView 8 was used as background.

During AHE, the increase of GM and WM volume was clearly more pronounced when compared to PHE (Figure 2). Significant bilateral increases of WM and GM volume were found in the frontal lobe, temporal lobe, occipital lobe as well as in the limbic lobe after AHE.

In addition, the frontal lobe, parietal lobe, temporal lobe, the anterior lobe and posterior lobe of the cerebellum and the sub-lobar region showed significantly increased ADC values when compared to baseline measurements. No areas with decreased regional GM and WM volumes or decreased ADC values could be observed during AHE. Subjects with and without AMS or with and without headache did not differ regarding GM and WM volumes or ADC values. Tables 5 and 6 and Figure 2 summarize the findings for the whole group caused by AHE.

**Table 5:** Anatomical regions with significant gray (GM) and white matter (WM) volume increases during the active hypoxic exposure (AHE) (p<0.001, uncorrected).

Cerebral region		Matter	Side	Cluster size mm3	MNI coordinates	t	Z-score
**Cerebrum**							
Frontal Lobe	Medial Frontal Gyrus	WM	R	9236	16.5 51 21	6.66	4.61
	Postcentral Gyrus	WM	R	85	46.5 −21 28.5	6.41	4.51
	Middle Frontal Gyrus	WM	L	85	−30 45 16.5	4.62	3.67
Temporal Lobe	Sub-Gyral	WM	L	961	−3.5 6 −37.5	6.11	4.39
	Sub-Gyral	WM	L	168	−46.5 −4.5 −16.5	4.93	3.83
	Superior Temporal Gyrus	WM	R	94	46.5 −49.5 9	4.80	3.77
Occipital Lobe	Lingual Gyrus	GM	L	685	−3 −99 −22.5	6.15	4.40
	Cuneus		R	458	19.5 −96 13.5	5.03	3.88
Limbic Lobe	Parahippocampa Gyrus	GM	R	129	27 −3 −18	5.25	3.99
	Anterior Cingulate	WM	L	171	−11 38 −6	4.89	3.81

Gray matter (GM), white matter (WM), Left (L), Right (R), coordinates (x, y, z) are given in Montreal Neurological Institute (MNI) space showing the center of each cluster.

**Table 6:** Anatomical regions with significant increases of apparent diffusion coefficient (ADC) during the active hypoxic exposure (AHE) (p<0.001, uncorrected).

Cerebral region		Matter	Side	Cluster size mm^3^	MNI coordinates	t	Z-score
**Cerebrum**							
Frontal Lobe	Superior Frontal Gyrus	GM	R	688	40 52 28	9.26	5.46
	Inferior Frontal Gyrus	GM	R	747	62 8 32	8.85	5.34
	Medial Frontal Gyrus	GM	R	153	4 52 −4	6.87	4.69
Parietal Lobe	Inferior Parietal Lobule	WM	R	157	64 −28 28	8.04	5.10
Temporal Lobe	Fusiform Gyrus	GM	L	86	−30 −38 −24	5.76	4.23
	Superior Temporal Gyrus	GM	L	61	−66 −4 8	5.16	3.95
Sub-lobar	Third Ventricle		R	1093	0 −20 0	8.52	5.25
	Insula	WM	L	80	−40 14 −2	6.58	4.58
**Cerebellum**							
Anterior Lobe	Culmen		R	529	38 −48 −32	6.87	4.69
	Culmen		L	95	−46 −52 −26	6.24	4.44
Posterior Lobe	Declive		L	56	−32 −66 −30	5.75	4.23

Gray matter (GM), white matter (WM), Left (L), Right (R), coordinates (x, y, z) are given in Montreal Neurological Institute (MNI) space showing the center of each cluster.

## Discussion

The main findings of the present study were that an 8-hour resting exposure to normobaric hypoxia (PHE) equivalent to an altitude of 5500 m caused AMS in ten of 20 healthy volunteers (50%) and was associated with volume increases in GM and WM as well as increases in ADC, irrespective of the presence of AMS. When exercising in hypoxia, 70% of the participants develop AMS, which was not significantly different from PHE. However, the combination of hypoxia and exercise caused clearly higher GM and WM volume and ADC increases when compared to PHE, indicating augmented water accumulation in the extracellular space, again irrespective of the presence of AMS. To our knowledge, this is the first study that uses this approach (hypoxic exercise and voxel based morphometry) to investigate the relationship between AMS and cerebral edema formation.

### Prevalence of AMS

The prevalence of AMS during PHE is in accordance with the findings reported by Kallenberg et al. [3]. The authors exposed 22 subjects for 16 hours to normobaric hypoxia (F_i_O_2_ = 12%) corresponding to an altitude of 4500 m and found an AMS prevalence of 50%. Roach et al. [7] showed that exercising in acute hypobaric hypoxia (∼4800 m) caused AMS in 86% of participants (N = 7), which was significantly higher than the prevalence of AMS (14%) during resting conditions. In the present study the difference in the AMS prevalence between PHE (50%) and AHE (70%) did not reach statistical significance. These divergent results are most likely attributable to the different ways of simulating high altitude (normobaric vs. hypobaric hypoxia), although previous findings [16] argue against a lower resting prevalence of AMS during hypobaric hypoxia.

### Magnetic resonance findings

During PHE we found slightly increased ADC values in various brain regions which are indicative for minor extracellular (vasogenic) edema [17]. This hypoxia-induced extracellular water accumulation was not related to AMS. Our findings are in accordance with two recently published MRI studies also demonstrating increased ADC values in all subjects exposed to hypoxia, irrespective of the presence of AMS [3], [6]. In addition, the comparison of the T1-weighted data sets revealed volume increases in GM and WM of different brain regions, also indicating spreading of the extracellular space caused by vasogenic edema. Short-term changes in cerebral volume are commonly caused by changes in tissue water content or perfusion. For example, a previous study showed mid-term brain volume changes in patients with hepatic encephalopathy due to therapy and volume increases were considered cerebral edema [18]. It has been suggested that the formation of mild extracellular brain edema subsequent to an increased permeability and/or a disruption of the blood brain barrier (BBB) tight junctions reflects the “normal” sequence of events that occur in the brain when a person is exposed to hypoxia [2]. Both, mechanical factors and biochemical mediators, that serve to maintain oxygen homeostasis, may contribute to an increased BBB leakage [5]. Several studies using the Kety-Schmidt technique [19], Xenon-133 technique [20] or Doppler sonography of the middle cerebral artery [21] demonstrated an enhanced cerebral blood flow (CBF) during acute hypoxia that however, seems to return to baseline values after several weeks of acclimatization at high altitude [22]. Additionally, several authors suggested that cerebral autoregulation (CA) might be impaired in all individuals exposed to hypoxia, irrespective if they develop AMS or stay healthy [4], [23]–[26], resulting in elevated cerebral capillary pressure, mechanical BBB disruption and vasogenic edema [27]. Bailey et al. [28] also provided convincing evidence for impaired CA subsequent to increased free radical formations during acute hypoxia. By contrast, they did not find any signs of BBB failure as indicated by the lack of change of S100β [28], [29], which argues against vasogenic edema formation subsequent to impaired CA and compromised BBB integrity. Apart from those mechanical responses to hypoxia, high altitude induces the expression of hypoxia inducible factor-1ά (HIF-1ά), which is responsible for the transcriptional activation of the vascular endothelial growth factor (VEGF) [30]. An increased production of VEGF following hypoxia is known to encourage the breakdown of the BBB [31], [32] via synthesis or release of nitric oxide (NO) [33]. Recent evidence suggests that AMS resistance is accompanied by increased levels of interleukin-1 receptor agonist (IL-1RA), heat shock protein (HSP)-70 and adrenomedullin, which are known to have anti-inflammatory and/or anti-permeability effects improving BBB function [34]. These findings indicate that AMS- subjects may be protected against BBB disruption and vasogenic edema, which argues against our findings that all subjects exposed to hypoxia develop mild vasogenic edema. However, without the determination of appropriate biomarkers of BBB leakage like S100β, this conclusion remains speculative. In light of our results we suggest, that mechanical and/or biochemical forces might increase cerebral capillary hydrostatic pressure and promote extracellular (vasogenic) edema subsequent to BBB disruption [2], which occurs irrespective of the presence of AMS.

While PHE caused only minor cerebral changes, GM and WM volume and ADC increases due to AHE were clearly more pronounced indicating augmented water accumulation in the extracellular space in these conditions. Mechanical and/or biochemical forces that alter BBB permeability seem to be elevated during exercise in hypoxia when compared to resting conditions. This assumption is supported by Subudhi et al. [35] and Vogiatzis et al. [36] who recently demonstrated that CBF is increased during sub-maximal exercise in hypoxia compared to resting conditions. This observation is most likely attributable to the cerebral vasodilatation caused by the hypoxic stimulus during submaximal exercise overriding the hypocapnia-induced vasoconstriction [37], [38]. However, despite this cerebral hyper-perfusion, oxygen delivery cannot be maintained when subjects exercise in hypoxia resulting in a dramatic decline of cerebral oxygen saturation as previously shown by Saito et al. [39]. They studied ten trekkers at 3700 m during moderate-intensity stepping exercise in hypoxia and registered a cerebral desaturation of 48% when compared to resting values. Further, recent evidence suggests that the activation of HIF-1 and VEGF is related to the severity of hypoxia [32], [40]. Thus, it could be argued that mechanical and biochemical forces are increased during AHE leading to an elevated BBB leakage, which would explain our findings indicating an increased formation of vasogenic brain edema during AHE. The fact that the left hemisphere is mainly activated during exhausting exercise is well known and was previously shown by Powell [41]. Therefore, we conclude, that the degree of hypoxemia plays a significant role in the development of hypoxia-induced vasogenic brain edema, which seems to be the typical response when individuals are exposed to or exercise in hypoxia.

Nevertheless, no differences in T_1_ or diffusion weighted images could be identified between subjects with and without AMS in the present study. This observation is in contrast to the findings of Kallenberg et al. [3] and Schoonman et al. [6] who both reported that AMS+ subjects exhibited decreased ADC values indicating intracellular (cytotoxic) edema. This has been attributed to a decreased activity and/or expression of the Na+/K+ - ATPase and a down-regulation of the cell membrane ionic pumping due to severe cerebral hypoxemia, leading to P_a_O_2_ values causing anaerobic glycolysis and a reduction of the cerebral metabolic rate of oxygen (CMRO_2_) [3], [6]. However, a recent positron emission tomography (PET) study of patients with acute ischemic stroke demonstrated that ADC values are poor predictors of reduced CMRO_2_ levels during severe hypoxia [42]. Further, both studies failed to provide any evidence for additional edema or brain swelling. Thus, it might be argued that the ADC decrease may simply reflect a “fluid re-distribution” from the extracellular space without any additional increases in brain volume or intracranial pressure [2]. However, whether such a minor shift of water into the cells of the corpus callosum may actually lead to the symptoms of AMS remains unknown [2]. In view of these results we suggest that the formation of extracellular edema reflects the “normal” sequence of events occurring in the brain when a person is exposed to hypoxia, which is even more pronounced when physical exercise is performed. However, we cannot support the assumption that AMS is an early, sub-clinical form of HACE and that both conditions share a common pathophysiology. Due to effectiveness of cyclooxygenase inhibition in the prevention and treatment of HAH [43], future studies should also focus on prostaglandin mediated inflammatory mechanisms, which might contribute to the enhanced nociception in the development of HAH and AMS [44].

### Study limitations

Several limitations of the present study have to be mentioned. The major limitation arises from the relatively small sample size (N = 20). Although there were no statistically significant changes between PHE and AHE regarding the prevalence of AMS, the markedly higher susceptibility during AHE (50% vs. 70%) is not negligible and may be of clinical importance. Therefore, a type II error cannot be excluded. Furthermore, the normobaric hypoxia applied in the present study does not reflect natural hypoxia which is characterized by reduced barometric pressure, cold, radiation, etc. Due to the fact that we did not simulate these environmental stress factors, we cannot be sure whether our results are also applicable to hypobaric conditions. In addition, a 3.0 Tesla magnetic scanner would have provided a higher resolution and signal-to-noise ratio making the MR investigation more sensitive for detecting cerebral abnormalities than a 1.5 Tesla scanner, used in the present study. However, due to the fact that our findings largely agree with those of other studies [3], [6], we do not have the impression that the use of a 1.5 Tesla scanner has biased our results. Finally, prior to the second MR imaging subjects were not kept hypoxic for several minutes. However, this short normoxic period did not affect AMS symptoms and should not have had any effects on brain edema formation. Among the particular strengths of the present study is the randomized, cross over study design and the application of exercise to exacerbate the hypoxic stress. This approach ensures the investigation of different degrees of hypoxia within the same subjects, thereby avoiding individual variations such as brain and ventricle size or genetic predisposition. Further, MR image processing and analyzing using voxel based morphometry has the advantage that regions of interest do not have defined a priori, which enables the investigation of cerebral changes of the entire brain.

### Conclusion

In conclusion, our findings provide further evidence that an exposure to normobaric hypoxia results in extracellular water accumulation, which is more pronounced when the hypoxic stress increases such as during exercise. This extracellular (vasogenic) edema formation was present in all subjects exposed to hypoxia, irrespective of the presence of AMS. We did not detect any indication of cytotoxic (intracellular) edema. Thus, we were not able to confirm our hypothesis that brain swelling and consequently AMS severity is more pronounced when subjects exercise in hypoxia compared to resting conditions. Consequently, these findings indicate that cerebral edema formation in acute hypoxia is not related to AMS development and that AMS and HACE are unlikely to share a common pathophysiological mechanism.
